# Evidence tiers for strain‐resolved long‐read metagenomics

**DOI:** 10.1002/imo2.70130

**Published:** 2026-08-09

**Authors:** Yanhua Han, Yunjuan Peng, Minghui Li, Xiaoyuan Wei, Jianmin Chai, Jiangchao Zhao, Ying Li, Feilong Deng

**Affiliations:** ^1^ School of Animal Science and Technology Foshan University Foshan China; ^2^ College of Animal Science South China Agricultural University Guangzhou China; ^3^ Department of Internal Medicine, Morsani College of Medicine University of South Florida Tampa USA

## Abstract

Long‐read metagenomics links variants, repeats, and mobile elements across individual molecules and can reveal lineage turnover that is obscured at the species level. The strength of the resulting inference, however, depends on the type and genomic span of the recovered linkage. We distinguish four evidence units—strain profiles, local haplotype blocks, genome‐scale strain candidates, and host‐linkage evidence—and describe the diagnostics appropriate to each. Confidence is reported separately as candidate, supported, or high confidence in context, based on read assignment, allele coherence, coverage continuity, boundary support, and independent confirmation. Ambiguous placement, contamination, consensus collapse, coverage breaks, reference bias, or unresolved linkage should prompt more conservative terminology. Aligning terms with the available evidence reduces overstatement in claims of strain reconstruction, host assignment, persistence, replacement, and engraftment, while making analyses easier to compare and review.

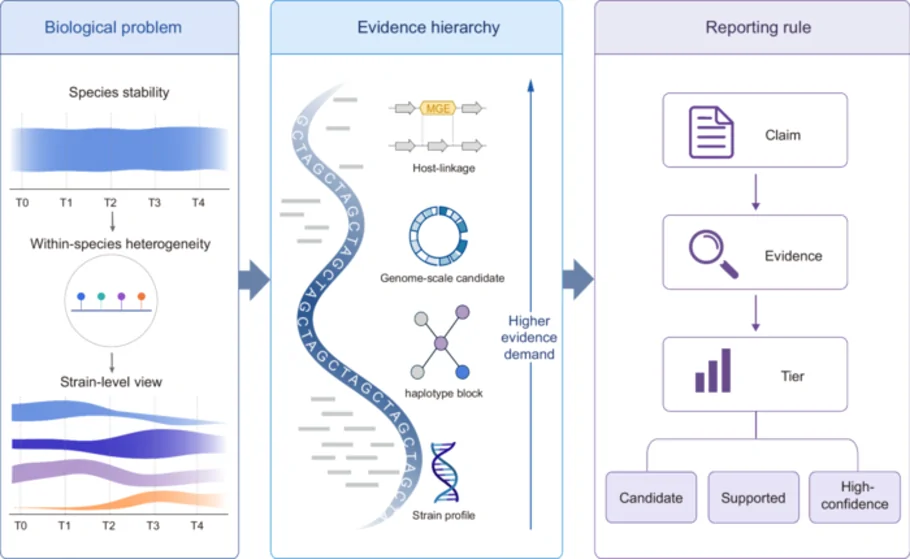

Metagenomic analyses increasingly move beyond species‐level abundance estimates to questions about strain composition and dynamics. A community may appear stable at the species level even as its constituent lineages change. After fecal microbiota transplantation, for example, a post‐treatment species profile that resembles the baseline profile may nevertheless reflect replacement of a resident lineage by a donor‐derived lineage [1] (Figure 1A). This distinction matters because the biological interpretation of an antimicrobial resistance gene, prophage, or plasmid‐like region depends on the lineage that carries it, not simply on the species label assigned to the reads.

**Figure 1:**
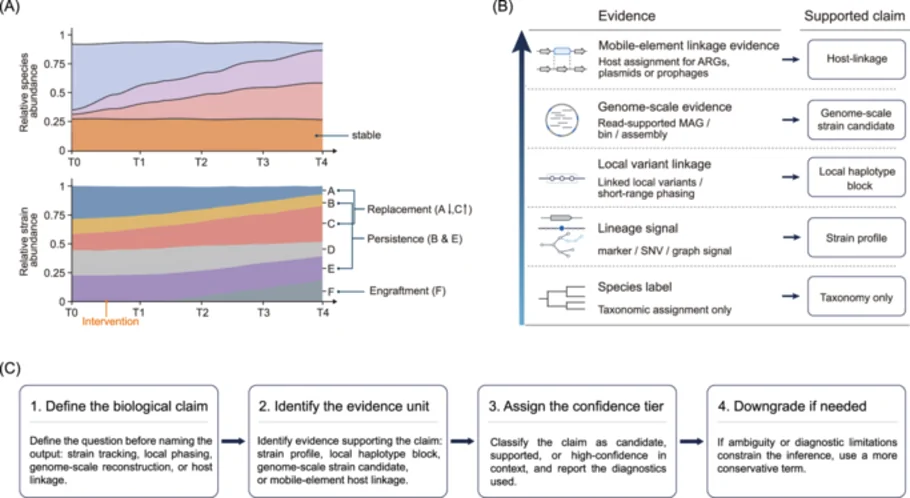
Strain‐level inferences begin with a species assignment but depend on distinct evidence units and an evidence‐aware workflow. (A) Species‐level abundance may remain stable despite changes in lineage composition. In this schematic longitudinal example, the species profile is similar across time points, whereas the lineage‐level view distinguishes replacement, persistence, and engraftment. T0 denotes the pre‐intervention baseline, and T1–T4 denote successive post‐intervention sampling time points. Letters A–F are schematic identifiers for six distinct strain‐level lineages within the focal species rather than taxonomic names. Replacement is the decline of one lineage accompanied by the expansion of another under the same species label; persistence is the continued detection of the same lineage over time; and engraftment is the establishment of a lineage that was absent or rare before intervention. Colors denote lineages. (B) Different strain‐level inferences require different forms of evidence. Taxonomic assignment supports a species label; signals based on marker genes, single‐nucleotide variants, or graph placement support a strain profile; and linked local variants support a local haplotype block. Read‐supported MAGs, bins, or assemblies can support a genome‐scale strain candidate when consistency checks are satisfied. Claims that link antimicrobial resistance genes, plasmids, or prophages to hosts require evidence such as boundary‐spanning reads, supported graph paths, or proximity‐ligation contacts. The arrangement reflects differences in genomic span and diagnostic requirements; these evidence units represent distinct claim types rather than stages in a single hierarchy. (C) A four‐step workflow for evaluating strain‐level claims. First, define the biological question before naming the analytical output. Second, identify the evidence unit that directly supports the claim. Third, assign the claim as candidate, supported, or high confidence in context and report the supporting diagnostics. Fourth, use a more conservative term when ambiguity or diagnostic limitations constrain the inference. Abbreviations: ARG, antimicrobial resistance gene; MAG, metagenome‐assembled genome; SNV, single‐nucleotide variant.

In metagenomic data, however, a strain is inferred rather than observed as a cultured isolate. Available methods recover different forms of strain‐level information, including marker‐based or single‐nucleotide variant (SNV)‐based profiles [2], local haplotype blocks, genome‐scale strain candidates, and signals linking mobile elements to hosts. These outputs differ in both genomic span and evidentiary requirements. Long reads improve strain resolution by linking variants across repeats and mobile‐element boundaries, but partial linkage can still be overinterpreted. A circular metagenome‐assembled genome (MAG), for example, should remain a candidate when allele mixtures, graph ambiguities, contamination, or unsupported junctions are present. Similarly, a local haplotype block does not establish genome‐wide continuity, and assignment to the same species or bin does not by itself identify the host of a mobile element.

This Commentary focuses on the gap between analytical output and biological claim. We organize strain‐level results into four evidence units—strain profiles, local haplotype blocks, genome‐scale strain candidates, and host‐linkage evidence—and assess confidence independently of evidence type (Figure 1B). We use three confidence tiers: candidate, supported, and high confidence in context. Rather than prescribing universal numerical cutoffs, the framework asks authors to define the biological claim, name the evidence unit that supports it, report the relevant diagnostics, and use more conservative terminology when ambiguity, contamination, consensus collapse, coverage breaks, reference bias, or unresolved linkage remains. This approach is intended to make strain‐level conclusions more precise, comparable, and easier to evaluate across analytical workflows.

## 1 MATCH TERMINOLOGY TO THE EVIDENCE

Overstatement often arises when these four evidence units are treated as interchangeable. In metagenomic datasets, “strain” denotes an inferred lineage rather than the cultured isolate used in classical microbiology. A strain profile is typically derived from marker genes, SNVs, graph paths, or database assignments. Marker‐based profilers and methods based on species‐level genome bins can generate reproducible profiles, including for uncharacterized species, but those profiles remain defined by markers or graph placement unless physical linkage or genome‐scale reconstruction is demonstrated [2, 3, 4, 5]. They are well suited to cohort‐level tracking and relative comparisons, but they do not by themselves constitute a reconstructed genome.

Local haplotype blocks provide linkage across a defined genomic interval. Long reads, assembly‐graph paths, or local assembly can show that variants occur together, that a minor lineage persists after intervention, or that a structural variant belongs to a particular local background. Graph‐based methods can also identify sequence segments whose abundance changes across related samples [6, 7, 8]. Unless these intervals can be connected across the genome, they are best described as local haplotypes, graph paths, or structural‐variant signals. Genome‐scale reconstruction requires broader support and may be pursued through assembly‐first workflows in which long reads are assembled, binned, remapped, and checked for consistency [9, 10, 11]. Even then, “candidate” remains an important qualifier: MAG quality tools estimate completeness and contamination but do not establish allele coherence or strain purity [12].

Host‐linkage claims require evidence that directly connects the element to the proposed microbial background [13, 14, 15]. Figure 1B separates taxonomic labels from the four strain‐level evidence units and summarizes the diagnostic requirements associated with each. Terminology should therefore reflect the genomic scale at which linkage has actually been demonstrated.

## 2 ASSIGN THE CONFIDENCE TIER

No single numerical threshold is appropriate for every microbial community. Effective read accuracy and effective span jointly determine how much strain‐resolving information a long‐read dataset contains (Figure S1A). The usable depth required per lineage depends on within‐species divergence and on the number of coexisting, closely related strains (Figure S1B). DNA extraction, sequencing depth, size selection, host depletion, multi‐sample design, high‐throughput chromosome conformation capture (Hi‐C), and hybrid sequencing can determine which evidence units can be reported defensibly (Figure S1C). Confidence tiers can nonetheless be assigned from the available diagnostics. A candidate claim identifies an analytical signal and provides basic quality information without implying full resolution. For a strain profile, authors should specify the marker set, graph, or database; for a MAG, they should report completeness and contamination estimates. A putative host link should remain at the candidate tier unless supported by boundary‐spanning reads or proximity‐ligation evidence.

At the supported tier, a tool‐assigned label must be accompanied by diagnostics that directly support the inference. For genome‐scale strain candidates, relevant evidence includes read remapping, coverage breadth and continuity, circularization or junction support where applicable, bin‐quality metrics, and tests for taxonomic discordance. Lineage‐defining variants should be concordant across reads, graph paths, or samples rather than forming incompatible mixtures. Profile‐level reports should describe read assignment, the available references or graph paths, the treatment of ambiguous placements, and the recurrence of the signal across samples. Host‐linkage claims require molecules or other data that connect the element to the proposed host background. Co‐assignment to the same species, bin, or genomic neighborhood can be informative, but it is weaker than a resolved host link.

Three diagnostics recur across the evidence units and should be defined explicitly. Coverage continuity refers to support that extends across informative regions rather than appearing only in isolated reads separated by unsupported intervals. Allele coherence refers to agreement among lineage‐defining variants across reads, graph paths, or samples, rather than incompatible mixtures. Boundary support requires reads or graph paths to span the junction being interpreted, such as a circularization edge, a prophage boundary, or the connection between an antimicrobial resistance gene and its proposed host. These criteria are intended to guide reporting, not to impose universal numerical cutoffs; the corresponding plots or summaries should accompany the claim. The “high confidence in context” tier should be reserved for conclusions supported by independent lines of evidence, such as replicate extractions, mock‐community benchmarks, cultured isolates, targeted assays, proximity ligation, longitudinal recurrence, or agreement across methods. Plausible alternatives should also be stated. Figure 1C summarizes the workflow, and Table S1 expands it into a reviewer checklist.

## 3 TWO ROUTES AND DOWNGRADE TRIGGERS

Assembly‐first workflows use a reconstructed genome as the primary evidence unit. Long‐read assembly can improve contiguity and recover gene neighborhoods, repeats, and long‐range associations around mobile elements [9, 10, 11]. Tools such as hifiasm‐meta, metaMDBG, and nanoMDBG show how long‐read assembly graphs can yield genome‐scale strain candidates, and comparisons with isolate genomes indicate that MAGs assembled from high‐fidelity long reads can approach isolate‐level accuracy in selected settings [16]. This route is summarized in Figure 2A. Its main vulnerability is that graph simplification, binning, or circularization may merge related lineages or close an assembly across unresolved repeats. Recent work has documented chimeras, premature circularization, haplotyping errors, excessive repeats, and phantom sequences in long‐read metagenome assemblies [17].

**Figure 2:**
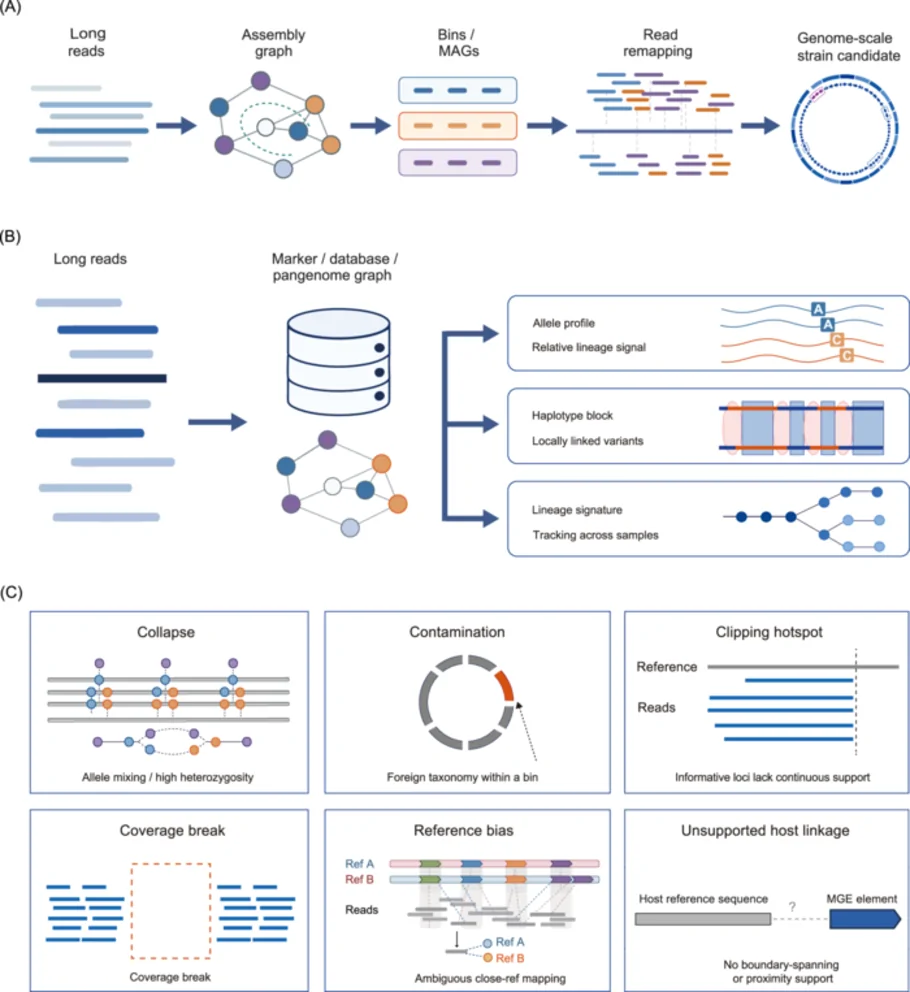
Two analytical routes and common reasons to downgrade strain‐level claims. (A) Assembly‐first workflows use long reads to construct assembly graphs, bins, or MAGs, followed by read remapping and consistency checks. They can yield genome‐scale strain candidates, but interpretation depends on graph ambiguity, bin contamination, allele coherence, coverage continuity, and boundary support. A complete‐looking or circular assembly remains a candidate unless its structure is supported by reads and appropriate diagnostics. (B) Read‐, graph‐, and database‐first workflows use marker sets, reference databases, or pangenome graphs to generate allele profiles, local haplotype blocks, or lineage signatures. These outputs can support tracking and comparison across samples, but they should not be described as whole‐genome strain reconstructions without genome‐scale linkage. (C) Consensus collapse, contamination, clipping hotspots, coverage breaks, reference bias, and unsupported host linkage can make genome‐scale or host‐linkage interpretations unreliable and should prompt more conservative terminology. Together, these diagnostics support assignment of an appropriate confidence tier. Abbreviations: MAG, metagenome‐assembled genome; MGE, mobile genetic element; Ref, reference.

Read‐, graph‐, and database‐first workflows produce profiles, graph paths, structural‐variant signals, or local haplotype blocks rather than reconstructed genomes. Examples include MetaMaps‐style read assignment, graph‐based structural‐variant detection, DESMAN and STRONG strain deconvolution, and pangenome‐graph profiling with PanTax [3, 4, 18, 19] (Figure 2B). These results can support tracking and comparison when the reference space and treatment of ambiguity are reported. Their principal limitation is reference dependence: if a relevant genome is absent, a novel lineage may be assigned to the closest known strain; if the database contains many near‐duplicate entries, reads from one lineage may be split across them. Unless genome‐scale linkage is demonstrated, the result is more accurately described as a profile, path, or local block.

Host assignment is subject to similar constraints. Long reads may link a prophage, a plasmid fragment, or an antimicrobial resistance gene to neighboring sequences, but that neighborhood does not always identify a resolved host genome. Apparent host associations can arise from a shared or chimeric contig, binning errors, correlated abundance, or unfiltered proximity‐ligation contacts. Evidence based only on co‐assignment to the same species, bin, contig neighborhood, or abundance pattern should therefore remain at the candidate tier. A stronger host‐linkage claim requires direct or orthogonal evidence—for example, boundary‐spanning reads, supported graph paths, or carefully filtered proximity‐ligation contacts—together with taxonomic and coverage patterns consistent with the proposed host. Claims about transfer, expression, or function require separate evidence beyond physical context.

Several failure modes are not apparent from summary quality metrics. Consensus collapse can produce a clean‐looking sequence that combines two coexisting lineages, and a MAG may pass completeness checks despite retaining mixed alleles. In low‐biomass or plate‐based studies, contamination can also create recurrent strain signals [20]. Figure 2C summarizes the main reasons to use more conservative terminology: consensus collapse, contamination, clipping hotspots, coverage breaks, reference bias, and unsupported host linkage. The figure is not intended to rank tools; it identifies points at which the biological interpretation should be reconsidered during manuscript preparation.

## 4 APPLYING THE FRAMEWORK IN MANUSCRIPTS AND PEER REVIEW

For each strain‐level result, authors should report the evidence unit, confidence tier, key diagnostics, and unresolved uncertainty. A local haplotype block, for example, can be considered supported when long reads link variants across a locus even if repeats prevent that linkage from extending across the genome. Claims based on reference databases, assemblies, or host‐linkage analyses should likewise specify how ambiguous assignments were handled, show the relevant remapping or coverage diagnostics, and state the limits of the recovered linkage.

Reviewers need not require complete genome resolution for every strain‐level analysis. Local haplotype blocks can support locus‐specific interpretation; strain profiles can support cohort‐level comparisons; and genome‐scale candidates can support analysis of gene neighborhoods when the reported diagnostics match the intended claim. When terminology implies a stronger inference than the data justify, the appropriate response is usually to use a lower‐resolution term, add the missing diagnostics, or present the result as qualified rather than central.

Long‐read metagenomics expands the range of strain‐level features that can be observed, but it does not eliminate uncertainty. Each result should be named at the scale supported by its evidence, with confidence stated separately.

## AUTHOR CONTRIBUTIONS

**Yanhua Han:** Writing – original draft; visualization. **Yunjuan Peng:** Writing – original draft; visualization. **Minghui Li:** Writing – original draft. **Xiaoyuan Wei:** Writing – review and editing. **Jianmin Chai:** Writing – review and editing. **Jiangchao Zhao:** Writing – review and editing; funding acquisition. **Ying Li:** Supervision; writing – review and editing; funding acquisition. **Feilong Deng:** Conceptualization; writing – review and editing; funding acquisition. All authors read and approved the final manuscript.

## CONFLICT OF INTEREST STATEMENT

The authors declare no conflicts of interest.

## AI STATEMENT

ChatGPT (GPT 5.5, OpenAI) was used only for English‐language editing. The authors verified the accuracy of all content and take full responsibility for the manuscript.

## ETHICS STATEMENT

No animals or humans were involved in this study.

## Supporting information

The online version of this article contains one supplementary figure and one supplementary table.

**Table S1:** Reviewer checklist for strain‐level claims.

**Figure S1:** Design space for strain‐resolved long‐read metagenomic claims.

## Data Availability

No new data or code were generated or analyzed in this study. Supplementary materials (figures, tables, graphical abstract, slides, videos, Chinese translated version, and updated materials) may be found in the online DOI or iMeta Science http://www.imeta.science/imetaomics/.
