# Supplementary material for: Evidence tiers for strain‐resolved long‐read metagenomics
Source: IMetaOmics. 2026 Aug 9;3(3):e70130. doi: 10.1002/imo2.70130 (PMC13674440; doi:10.1002/imo2.70130)
Supplement: Supplementary file 1 [file IMO2-3-e70130-s002.docx]

**Supporting Information to**

**Evidence tiers for strain-resolved long-read metagenomics**

**Running title:** Evidence tiers in long-read metagenomics

Yanhua Han^1,#^, Yunjuan Peng^2,#^, Minghui Li^1,#^, Xiaoyuan Wei^3^, Jianmin Chai^1^, Jiangchao Zhao^2^, Ying Li^1,*^, and Feilong Deng^1,*^

^1^ School of Animal Science and Technology, Foshan University, Foshan, 528225, China

^2^ College of Animal Science, South China Agricultural University, Guangzhou, 510642, China

^3^ Department of Internal Medicine, Morsani College of Medicine, University of South Florida, Tampa, 33612, USA

^#^These authors contributed equally: Yanhua Han, Yunjuan Peng, and Minghui Li

^*^Correspondence: fdeng@fosu.edu.cn (Feilong Deng); yingli@fosu.edu.cn (Ying Li).

**SUPPLEMENTARY FIGURES**

**Figure S1. Design space for strain-resolved long-read metagenomic claims.**

(A) Effective accuracy and effective span jointly define the broad information available from a long-read metagenomic dataset. HiFi and ONT are shown as partly overlapping practical regimes rather than as a fixed ranking of sequencing platforms. HiFi commonly provides low-indel evidence useful for allele-level inference and genome recovery, whereas modern ONT datasets, including high-accuracy or duplex-enabled modes when used, can provide longer-range linkage together with improved accuracy. The exact position of any dataset depends on DNA quality, read-length distribution, sequencing chemistry, basecaller, polishing, and filtering. (B) Required sequencing depth should be interpreted at the lineage level rather than only at the sample level. As within-species variation and the number of coexisting closely related strains increase, more usable per-lineage depth is needed to distinguish lineages, link informative variants, and avoid consensus collapse. The curves illustrate relative evidence demand, not numerical thresholds. A sample can therefore have high total depth but still provide insufficient support for a minor lineage or a genome-scale strain claim. (C) Experimental design determines which evidence units can be defensibly claimed. High-molecular-weight DNA, sequencing depth, size selection, host depletion, multi-sample design, Hi-C or other proximity-ligation support, and hybrid sequencing can increase usable depth, span, or independent linkage evidence. These levers do not automatically make a dataset strain-resolved; they shift what can be safely reported from variant-level signals to local haplotype blocks or genome-scale strain candidates, depending on the evidence available. The figure is intended as a planning guide for matching biological questions to evidence units, not as a benchmark or platform ranking. Abbreviations: DNA, deoxyribonucleic acid; Hi-C, high-throughput chromosome conformation capture; HiFi, high-fidelity; HMW, high-molecular-weight; MAG, metagenome-assembled genome; ONT, Oxford Nanopore Technologies; Q20, a Phred quality score of 20; R10.4.1, Oxford Nanopore Technologies R10.4.1 pore chemistry.
